# Compensatory Relearning Following Stroke: Cellular and Plasticity Mechanisms in Rodents

**DOI:** 10.3389/fnins.2018.01023

**Published:** 2019-01-31

**Authors:** Gustavo Balbinot, Clarissa Pedrini Schuch

**Affiliations:** ^1^Brain Institute, Universidade Federal do Rio Grande do Norte, Natal, Brazil; ^2^Graduate Program in Rehabilitation Sciences, Universidade Federal de Ciências da Saúde de Porto Alegre (UFCSPA), Porto Alegre, Brazil

**Keywords:** stroke, rehabilitation, motor learning, pharmacotherapy, plasticity

## Abstract

von Monakow’s theory of diaschisis states the functional ‘standstill’ of intact brain regions that are remote from a damaged area, often implied in recovery of function. Accordingly, neural plasticity and activity patterns related to recovery are also occurring at the same regions. Recovery relies on plasticity in the periinfarct and homotopic contralesional regions and involves relearning to perform movements. Seeking evidence for a relearning mechanism following stroke, we found that rodents display many features that resemble classical learning and memory mechanisms. Compensatory relearning is likely to be accompanied by gradual shaping of these regions and pathways, with participating neurons progressively adapting cortico-striato-thalamic activity and synaptic strengths at different cortico-thalamic loops – adapting function relayed by the striatum. Motor cortex functional maps are progressively reinforced and shaped by these loops as the striatum searches for different functional actions. Several cortical and striatal cellular mechanisms that influence motor learning may also influence post-stroke compensatory relearning. Future research should focus on *how* different neuromodulatory systems could act before, during or after rehabilitation to improve stroke recovery.

## Introduction

Recovery following stroke requires locating reorganization processes in the brain where the necessary motor and sensory signals converge. Once located, descriptions of mechanisms for optimization can provide neural substrates for recovery. In the cortex and striatum, waves of growth inhibiting and promoting factors related to cellular and plastic changes are triggered by the lesion. Cellular death leads to homeostatic destabilization and to a subsequent process of repair leading to partial recovery of cellular function (Carmichael, 2003). Cellular reorganization processes take place in brain regions adjacent or previously connected to the affected cells, such as: periinfarct tissue, cortico-cortical pathways, cortico-striatal pathways and cortico-thalamic pathways. Rehabilitation and pharmacological therapies are used to optimize and guide these cellular and plasticity changes. This complex recovery process following stroke involves several factors, here we suggest that cellular and plasticity mechanisms related to motor learning are likely active.

Stroke induces a permissive environment for axonal sprouting through growth-promoting proteins in the boundary zone to the ischemic core (Li et al., 1998; Carmichael et al., 2005). The permissive environment after stroke leads to cortical and corticospinal tract rewiring (Winship and Murphy, 2008; Wahl and Schwab, 2014) and other forms of long-term plasticity associated with learning (Hess and Donoghue, 1994; Hess et al., 1996). These changes associated with compensatory relearning are expected to produce both increases and decreases in synaptic strength distributed throughout the complex neural networks (bidirectional changes; Cohen and Castro-Alamancos, 2005). The precise homeostatic changes in order to refine synaptic connectivity and to adjust synaptic strengths to promote the stability needed for motor recovery following stroke are still poorly understood (Turrigiano and Nelson, 2004; Murphy and Corbett, 2009).

To enhance stroke recovery, the interaction of fundamental interconnected areas of research - such as motor learning and endogenous plasticity mechanisms, urgently requires new and innovative approaches (Krakauer, 2006; Krakauer et al., 2012). It has been 12 years since Krakauer suggested the relevance of motor learning for stroke recovery and rehabilitation (Krakauer, 2006). A brief review of recent clinical studies on stroke recovery highlights the positive effects of virtual reality for motor learning retention (Carregosa et al., 2018) and also in combination with transcranial stimulation (Fuentes et al., 2018) or exoskeletons (Grimm et al., 2016); and the prominent effects of constraint-induced movement therapy (CIMT) (reviewed elsewhere; Hatem et al., 2016). Motor skill relearning using CIMT is considered a promising and efficient method to improve clinical prognosis in stroke motor rehabilitation. Nevertheless, the authors highlight the need of more information about the integration of other motor skill learning techniques other than CIMT (Hatem et al., 2016). We suggest that using a reverse translation approach, preclinical stroke research may unveil the mechanisms involved in compensatory relearning to gauge the dose and timing of rehabilitation, drug therapy or the combination of both. For example, the use of pharmacotherapy in combination with physical rehabilitation before, during or after the rehabilitation section to maximize relearning effects. In this review, we merge principles and mechanisms of motor learning and stroke recovery and present a new perspective based on *where* and *how* compensatory relearning occurs.

New treatments for post-stroke impairments may depend on a better understanding of the neural mechanisms and influences of compensatory behavior (Jones, 2017). One of the challenges of understanding *how* compensatory relearning occurs is the fluid nature of memories, with participating brain regions dynamically shifting over time (Makino et al., 2016). To test if stroke recovery is mediated by relearning mechanisms, more studies with high temporal and spatial precision are needed (Makino et al., 2016). Recent findings suggest that the ‘stroke recovery circuit’ may parallel memory formation during learning tasks (Caracciolo et al., 2018); and that thalamo-cortical plasticity promotes stroke recovery (Tennant et al., 2017). These two examples highlight how temporal and spatial information needs to be integrated to unveil the mechanisms of compensatory relearning. The purpose of this review is to condense experimental findings of the large literature on motor skill learning and post-stroke recovery. We refer the reader to other recently published reviews for additional perspectives of cellular and plastic mechanisms of motor skill learning and stroke recovery. We also offer our perspectives on *how* to improve stroke recovery focused on compensatory relearning.

## The Cortical Circuit

The cellular and synaptic organization of the somatosensory cortex supports the primary motor neurons and the storage capability needed to encode movements (Penfield and Rasmussen, 1950; Jankowska et al., 1975; Nudo et al., 1996a; Hosp and Luft, 2011). Movements are represented in the motor cortex in regions related to forelimb or hindlimb responses (rostral forelimb area and caudal forelimb area; Neafsey and Sievert, 1982). At each region distinct cortical layers function to receive, integrate and transmit the motor output. The cortical circuit is shaped by redundancy that provides the flexibility needed to network changes, such as during post-stroke recovery (Sanes and Donoghue, 2000). For example, the rostral forelimb area is considered the putative premotor area in the rodent and is involved in post-stroke reorganization of motor representations (Neafsey and Sievert, 1982; Dancause, 2006; Dancause et al., 2015; Touvykine et al., 2016). This reorganization is tailored by thalamo-cortical loops, which are the building blocks of a homeostatic and functional movement network.

### Input Stage: Thalamo-Cortico-Thalamic and Thalamo-Cortico-Striatal Loops

At the input stage cortical principal cells receive, but also send information to the thalamus, integrating a redundant system that integrates sensory and motor signals (Haber and Calzavara, 2009; Leyva-Díaz and López-Bendito, 2013). Thalamo-cortical pathways receive higher-order information – already processed by other cortical or extra-cortical regions (Castro-Alamancos and Connors, 1997). For example, thalamo-cortical projections from the basal ganglia terminate in cortical layers I/II, III/IV, and V (McFarland and Haber, 2002); but also from the sensory periphery that projects sparsely to layers V and I (Castro-Alamancos and Connors, 1997). Cortico-thalamic axons are formed by corticofugal pyramidal neurons located in the cortical layer VI, and to a smaller extent layer V (Donoghue and Kitai, 1981; Leyva-Díaz and López-Bendito, 2013) (except where noted, corticofugal, hereafter refers to cortical projections to descending pathways). Interestingly, cortico-thalamic neurons send information that, after relayed by the thalamus, are redirected to a different cortical region integrating different cortical areas into a global network (Leyva-Díaz and López-Bendito, 2013).

As described above, deep layer cortical neurons send projections to thalamus, and thalamus projects back to the striatum and to the superficial, the middle, and the deep cortical layers (layers I/II, III/IV, and V) (Paré and Smith, 1996; McFarland and Haber, 2000, 2002). Thus, projections that terminate in deep cortical layers, e.g., layer V, are likely to form thalamo-cortico-thalamic and thalamo-cortico-striatal loops (Haber and Calzavara, 2009). In other words, deep layer projections may interact with neurons that project back to both the thalamus and striatum. As a result, this interaction can reinforce cortico-thalamic and cortico-striatal inputs to specific cortico-basal ganglia circuits and may be involved in the development of specific learned behaviors (McFarland and Haber, 2002).

Superficial cortical layers I/II also receive thalamo-cortical inputs and can control the activity of any neuron with apical dendrite ascending to layer I (Haber and Calzavara, 2009). Interestingly, due to the more widespread thalamo-cortical terminals in layer I, this input can affect adjacent cortical populations and cortico-cortical connections. Thereby, potentially modulating a different loop at a different cortical region (Haber and Calzavara, 2009). This type of plasticity can be of particular interest during stroke recovery and for compensatory relearning mechanisms. Given that after a cortical ischemia the infarct core is surrounded by functional tissue, it is likely that the surviving tissue can share some of the specific striatal-thalamo-cortical projections with the lesioned tissue. Thus, superficial cortical layers would participate of thalamo-cortico-thalamic and thalamo-cortico-striatal loops that would reinforce new connections during stroke recovery and compensatory relearning of motor tasks (Haber and Calzavara, 2009; Shmuelof and Krakauer, 2011).

**FIGURE 1:**
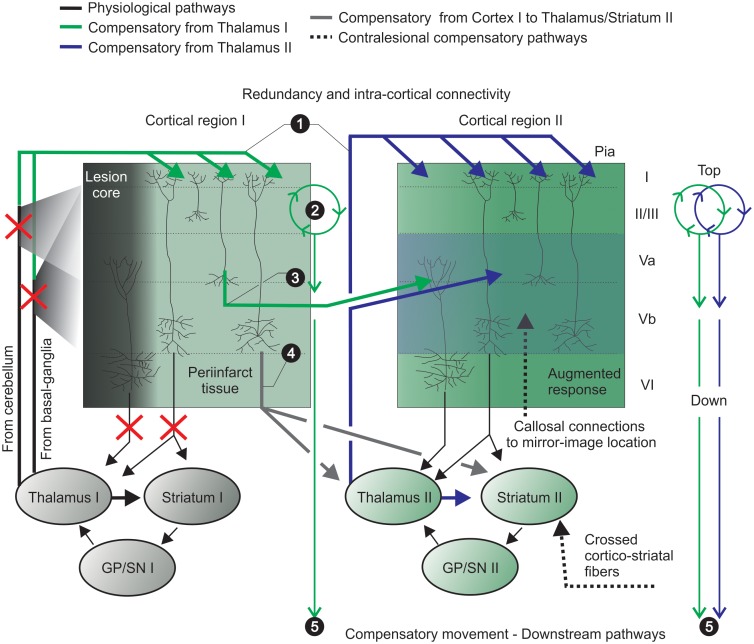
Pronounced intra-cortical connectivity and redundancy are remarkable features of the motor cortex. Motor cortex caudal (e.g., cortical region I) and rostral (e.g., cortical region II) forelimb areas contain the primary motor neurons that encode motor map representations of forelimb skilled movements. Pyramidal neurons project to brainstem and spinal cord (not shown) and send collaterals to striatum and thalamus – integrating thalamo-cortico-thalamic and thalamo-cortico striatal loops. (1) widespread thalamo-cortical connections common to both cortical regions (I and II) target superficial layers and reach damaged, periinfarct and spared areas (upper green and blue solid lines); (2) the preamplifier-like network (green circular loop) captures thalamic I signals and drives output neurons in lower layers (Weiler et al., 2008); (3) horizontal cortico-cortical connections of neurons receive and retransmit this indirect thalamic information (previously shared with the infarct core area) (green solid lines); (4) cortico-thalamic and cortico-striatal projections (from cortical region I to II; gray solid line) integrate another striatal-thalamo-cortical loop (cortical region II). The putative participation of crossed cortico-striatal and cortico-cortical fibers is shown (hashed black lines, bottom right). (5) Cells from adjacent/spared tissue (cortical region II) share thalamo-cortical inputs with interconnected/intertwined thalamo-cortical circuits of the stroke-disrupted network to control compensatory relearning of movements. GP, globus pallidus; SN, substantia nigra.

In other words, in the rodent agranular motor cortex, asymmetrical thalamic projections target layers I, II/III and V (from basal-ganglia) and layers II/III and V (from cerebellum) (reviewed in Peters et al., 2017). Stroke disrupts these thalamic targets but, the above-mentioned (1) widespread of thalamo-cortical inputs to superficial layers may affect adjacent spared cortical tissue. (2) Superficial layers may act as a preamplifier-like network, which captures these thalamic signals and drives output neurons in lower layers (Weiler et al., 2008). (3) Abundant cortico-cortical communication may affect adjacent and relevant cortical functional columns and integrate the process of augmented responses in deep layers supplemented by (4, 5) compensatory cortical, thalamic and striatal regions. These afferents projecting to layer V may initiate a sequence of synaptic and intrinsic membrane-dependent events, which prime the cortical network and lead to an augmented response due to heightened neuronal excitability in layer V and may favor compensatory relearning of movements (further explored in section “Endogenous Plasticity Mechanisms: Use-Dependent Plasticity, Augmented Responses and Neuromodulation”; Castro-Alamancos and Connors, 1996a) (Figure 1).

It is important to consider a few drawbacks of the layer specificity described here. First, that the existence of a layer IV in the rodent motor cortex is still under discussion and can be referred as the deep layer III or the superficial layer Va (Kaneko, 2013; Yamawaki et al., 2014). Second, it has been recently suggested that future studies should focus on specific circuits defined by functional cell type composition rather than the common oversimplification of laminar distribution (Guy and Staiger, 2017). Nevertheless, at the input level an abundant literature supports the importance of lamina-specific activity changes for motor learning (reviewed in Peters et al., 2017). Future studies should take advantage of the recent methods to reveal cell types and their changed post-stroke connectivity, with impact to novel stroke recovery mechanisms focused on compensatory relearning (similarly to striatal microcircuitry; Silberberg and Bolam, 2015).

### Output Stage: Redundancy and Intra-Circuit Connectivity

The output cells of the cerebral cortex are the pyramidal cells (70–80% of cortical neurons; Feldman, 1984). Their processing of sensorimotor inputs is beyond a simple output signal to descending motor neurons. The descending corticofugal pathway is complex with several intra- and extra-cortical collaterals and distinct terminations (Donoghue and Kitai, 1981). Primary projections are directed to the spinal cord, with secondary collaterals to the striatum, red nucleus, caudal pons and medulla (Donoghue and Kitai, 1981; Reiner et al., 2003; Leyva-Díaz and López-Bendito, 2013).

Pyramidal cells also show remarkable intra-circuit connectivity – intra-cortical synapses account for ≈70% of total synapses onto pyramidal cells (Amitai and Connors, 1995), both intra-layer (layer V; Markram et al., 1997) and inter-layers (layers II–V or VI–IV; Lund et al., 1993). Cortico-cortical connections are either within the same hemisphere (ipsilateral cortico-cortical connections) or from the opposite hemisphere (callosal connections) (Leyva-Díaz and López-Bendito, 2013). In rodents callosal pyramidal neurons are mainly at cortical layers II/III/IV (≈80%), layer V (≈20%) and, to a lesser extent, layer VI (Leyva-Díaz and López-Bendito, 2013). In addition, a population of layer V medium-sized pyramidal neurons is of cortico-striatal neurons that cross to the contralateral hemisphere (crossed cortico-striatal neurons; Wilson, 1987; Lévesque et al., 1996; Anderson et al., 2010).

The interconnection between hemispheres can lead to short- and long-term motor plasticity. The activation of motor neurons in the contralesional hemisphere can induce activation of cortico-cortical callosal projections to the ipsilesional hemisphere - in the same functional cortical column. For example, neurons from the contralesional caudal forelimb area can prime neurons in the spared ipsilesional caudal forelimb area (e.g., periinfarct region) (Castro-Alamancos and Connors, 1996a). Indeed, callosal cortico-cortical neurons extend axons to mirror-image locations in the same functional area at the contralateral hemisphere (bilateral integration of information; Greig et al., 2013). Ipsilesional neurons would also undergo plastic changes modulated by the newly formed cortico-striatal connections (crossed cortico-striatal neurons; Cheng et al., 1998) during rehabilitation.

In brief, at the output stage pyramidal cells integrate a complex cortical network to produce movement. Including abundant connections with the contralateral hemisphere. Plastic changes of callosal connections is thought as a mechanism underlying the physiological reorganization in the contralesional hemisphere following stroke (Dancause, 2006). In many ways the cortical circuitry is built with redundancy, this allows compensation by spared regions when a lesion occurs.

### Endogenous Plasticity Mechanisms: Use-Dependent Plasticity, Augmented Responses and Neuromodulation

Use-dependent plasticity plays a pivotal role on post-stroke functional recovery and on motor learning (Nudo et al., 1996b; Nudo et al., 1996a). For example, potentiation of thalamo- and cortico-cortical afferents by high frequency stimulation of the corpus callosum induces cortical LTP (Chapman et al., 1998) and increases forelimb motor representations, branch complexity, dendritic length and spine density in layer V (Monfils et al., 2004). In the motor cortex the induction of LTP is only possible with partial blockage of cortical GABA_A_Rs (Castro-Alamancos and Borrell, 1995). Suggesting that a fine tuning between excitation and inhibition is paramount to motor cortex use-dependent plasticity.

Under physiological conditions a strong glutamatergic afferent input from the motor thalamus innervates cortical pyramidal neurons (mainly layer V) (Amitai, 2001; Haber and Calzavara, 2009; Kuramoto et al., 2009). As aforementioned, these projections initiate a sequence of synaptic and intrinsic membrane-dependent events that prime the cortical network and induce an augmented response, i.e., heightened neuronal excitability (Castro-Alamancos and Connors, 1996a,c) (see Figure 1 – layer V augmented response). Interestingly, this augmented response of layer V is blocked by active exploration or skilled behavioral performance and induced by inactive behavioral states (Castro-Alamancos and Connors, 1996b). Suggesting a dynamic modulation of short-term thalamo-cortical plasticity, which can occur during and after motor skill relearning (Biane et al., 2016). Given that the spread of the augmenting response to upper cortical layers depends of synaptic interconnections and active dendritic conductance (Castro-Alamancos and Connors, 1996a), it is likely that after stroke the more permissive environment (Carmichael et al., 2005; Murphy and Corbett, 2009) could favor this short-term plasticity through horizontal pathways of layers II/III in M1 (Hess et al., 1996), reaching adjacent cortical tissue (e.g., cortical region II – Figure 1).

In brief, stroke recovery depends on cortical plasticity and this neuroplasticity is likely to require exploration of spared movements by the striatum (Shmuelof and Krakauer, 2011). We suggest that to unveil *how* these cortico-thalamo-cortical loops act on neuroplasticity mechanisms, a better understanding of the *in vivo* interactions during compensatory relearning is necessary. Previous evidence of *where* these changes occur are abundant, for example skilled training increase: dendritic length and arborization in layer II/III and V motor neurons (Greenough et al., 1985; Withers and Greenough, 1989; Kolb et al., 2008); number of synapses per neuron in layer V (hemisphere contralateral to the trained paw) (Kleim et al., 2004); dendritic arborization of cortical layer V in the contralesional motor cortex (Biernaskie and Corbett, 2001). The bulk of these findings suggests that callosal cortico-cortical, crossed cortico-striatal, and ipsilateral (uncrossed corticospinal projections; Vahlsing and Feringa, 1980) projections undergo neuroplasticity changes related to compensatory relearning of motor tasks.

#### Neuromodulation

**FIGURE 2:**
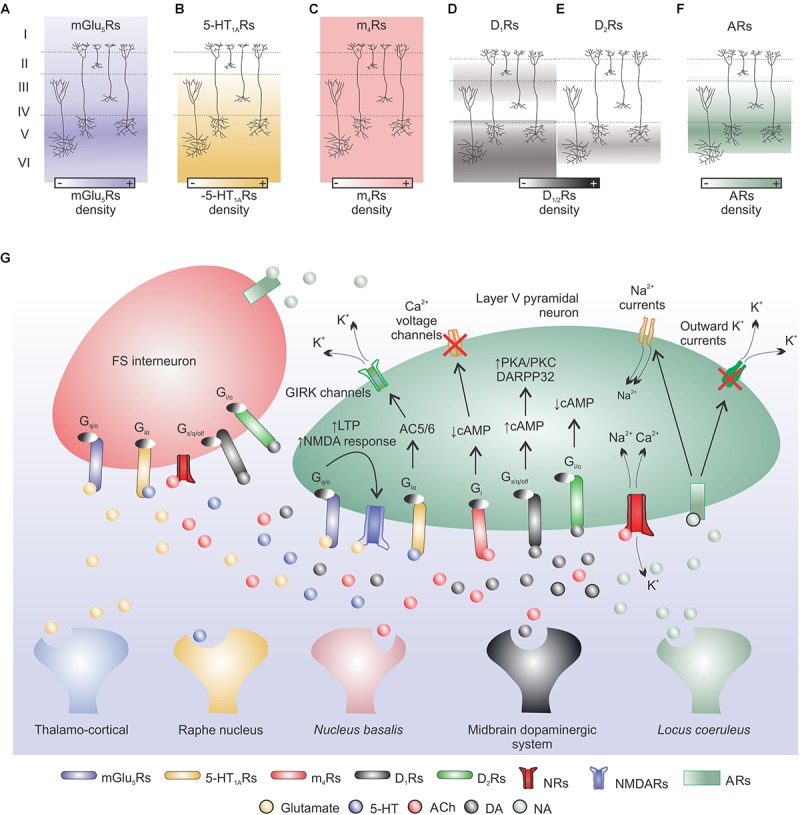
Neuromodulation: main cortical neurotransmitter systems involved in motor learning. **(A)** Glutamate release from cortical or thalamic afferents can modulate cellular excitability and short/long-term plasticity in cortical pyramidal neurons. Metabotropic glutamate receptors (mGlu_5_Rs) are mainly expressed in cortical layer V and act via G_q/O_ protein on downstream targets. This interaction can enhance NMDARs activity and induce LTP in pyramidal neurons. **(B)** Raphe nucleus serotonin (5-HT) can bind to 5-HT_1A_Rs (high mRNA expression in cortical layers V and VI) and via a G_iα_ protein-AC5/6 pathway induce K^+^ efflux leading to cell hyperpolarization, both in pyramidal cells and FS interneurons. **(C)** *Nucleus basalis* acetylcholine (ACh) binds to muscarinic receptors (MRs; e.g., m_4_Rs) or nicotinic receptors (NRs). m_4_Rs are expressed in all cortical layers and are coupled to G_i_ proteins that can reduce cellular activity through cAMP signaling. NMDARs are permeable to Na^+^, K^+^, and Ca^2+^ ions and are modulated by intra- and extra-Ca^2+^ concentrations (not shown). **(D,E)** Dopamine (DA) released by the midbrain dopaminergic system can bind to D_1_Rs (low expression in layers II–III and high expression in layers V–VI) or D_2_Rs (expressed in layer V but at a lower extent when compared to D_1_ expression) and increase or decrease cellular excitability, respectively, via cAMP acting on downstream targets (e.g., DARPP32). **(F)** *Locus coeruleus* noradrenaline (NA) released to the cerebral cortex binds to adrenoceptors (ARs) highly expressed in cortical layers IV and V. NA may increase cortical excitability via a reduction of outward K^+^ currents and increase of Na^+^ currents. **(G)** Simplified model of cortical neurotransmitter systems involved in motor learning.

Different types of metabotropic glutamate receptors (mGluRs) are expressed throughout the cortex (mGlu_1,2,3,5,7a/b,8a/b_), where mGlu_5_R has the strongest expression (Shigemoto and Mizuno, 2000; Ferraguti and Shigemoto, 2006) (Figure 2A); intense expression of mGlu_5_R was also detected in cortical GABAergic interneurons (Kerner et al., 1997). mGluRs provide a mechanism by which adjustments of fine-tune activity occurs at the same synapses of fast glutamatergic synaptic responses (Conn and Pin, 1997). Activation of mGlu_5_Rs can increase NMDA-evoked responses in the cortical tissue and, for example, mGlu_5_Rs antagonism can enhance MK-801 impairments of instrumental learning (Homayoun et al., 2004).

Cortical neurons also express several serotonin (5-HT) receptor subtypes (5-HT_1A/B,2A/C,3,4,6,7_; Celada et al., 2013), for example, cortical 5-HT_1A_Rs have high mRNA expression in cortical layers V and VI (Pompeiano et al., 1992; Puig et al., 2005; Singh and Staines, 2015) (Figure 2B). It is suggested that 5-HT modulation of motor cortex excitability leans toward facilitation (Singh and Staines, 2015). Interestingly, cortical 5-HT_1A_Rs activation has an overall excitatory effect on the neural networks that give rise to movement representations (Scullion et al., 2013). Selective serotonin reuptake inhibition improves learning and motor outcomes in animal models of ischemic stroke at different tasks, e.g., rotarod, staircase reaching, cylinder test, adhesive label test (McCann et al., 2014).

Another important cortical neuromodulator is acetylcholine (ACh), which can exert multiple effects on cortical neurons depending on type of target cell, pre- or post-synaptic receptor localization and receptor subtype (Wada et al., 1989, 1990; McGehee et al., 1995; Porter et al., 1999); resulting in a very complex modulation (Lucas-Meunier et al., 2003). Due to its diffuse cortical innervation some authors suggest a modulating rather than direct/synaptic role of ACh on the activity of the cortical circuitry (Lucas-Meunier et al., 2003 for references). Two major classes of ACh receptors are present in the rat cortex: muscarinic receptors (MRs) and nicotinic receptors. In the adult rat brain, cortical MRs subtypes are: m_1_ (26.4%), m_2_ (21.4%), m_3_ (7.7%), m_4_ (44.2%), and m_5_ (<1%) (Tice et al., 1996); in addition, ACh MRs are located at all cortical layers (Cortés and Palacios, 1986; Frey and Howland, 1992) and are metabotropic (Lucas-Meunier et al., 2003) (Figure 2C). Although there is a dense presence of cortical cholinergic receptors, the role of ACh on motor learning is still relatively underexplored. Conner’s group pioneer research showed that the basal forebrain cholinergic system is essential for cortical plasticity associated with motor learning (Conner et al., 2003). Later, they showed that cholinergic mechanisms are essential for cortical map plasticity after a skilled motor training (Ramanathan et al., 2009); and that cholinergic activation within the motor cortex modulates cortical map plasticity and motor learning (Conner et al., 2010). Interestingly, the basal forebrain cholinergic system is required for successful post-stroke rehabilitation, with direct impact on cell morphology (Wang et al., 2016).

Dopamine (DA) receptors are selectively expressed in different cortical layers. Motor cortex D_1_ receptors are expressed at superficial (low expression; layers II–III) and deep (high expression; layers V–VI) layers (Savasta et al., 1986) (Figure 2D). D_2_-receptors are expressed in cortical pyramidal layer V but at a lower extent when compared to D_1_ expression (Ariano et al., 1993; Awenowicz and Porter, 2002) (Figure 2E). D_1_ mRNA is present in cortico-cortical, cortico-thalamic and cortico-striatal neurons and D_2_ mRNA is restricted to layer V of cortico-striatal and cortico-cortical neurons (Gaspar et al., 1995). The overall effect of DA on cortical pyramidal cell excitability may depend on phasic changes in DA concentration and GABAergic inhibition tone (Gulledge and Jaffe, 2001). D_1_/D_2_ dopamine receptors activation is necessary for successful motor skill learning (Hosp et al., 2011). And the integrity of the dopaminergic mesencephalic-M1 pathway is also fundamental for motor learning in rats (Hosp and Luft, 2013). Suggesting that the M1 dopaminergic system is paramount to motor skill learning. DA system is also involved on cortical motor map representations (Brown et al., 2009, 2011), movement generation (Parr-Brownlie and Hyland, 2005) and LTP-like plasticity (Korchounov and Ziemann, 2011). Thus, DA has an important role on the modulation of intra-cortical excitability to enhance plasticity and to promote motor skill learning and execution.

Finally, adrenoceptors (ARs) are most present in cortical layers IV and V and the subtypes α_1A/B/D,2A/B/C/D_ and β_1,2,3_ are found in the cortical tissue (Wang and McCormick, 1993) (Figure 2F). Overall, noradrenaline (NA) increases the excitability of cortical pyramidal cells, but also the activity of cortical GABAergic non-pyramidal cells (Kawaguchi and Shindou, 1998). In the motor cortex, blockade of NA receptors suppresses the induction of LTP-like plasticity (Korchounov and Ziemann, 2011). Figure 2G shows a simplified model of cortical neurotransmitter systems involved in motor learning.

## The Striatal Circuit

**FIGURE 3:**
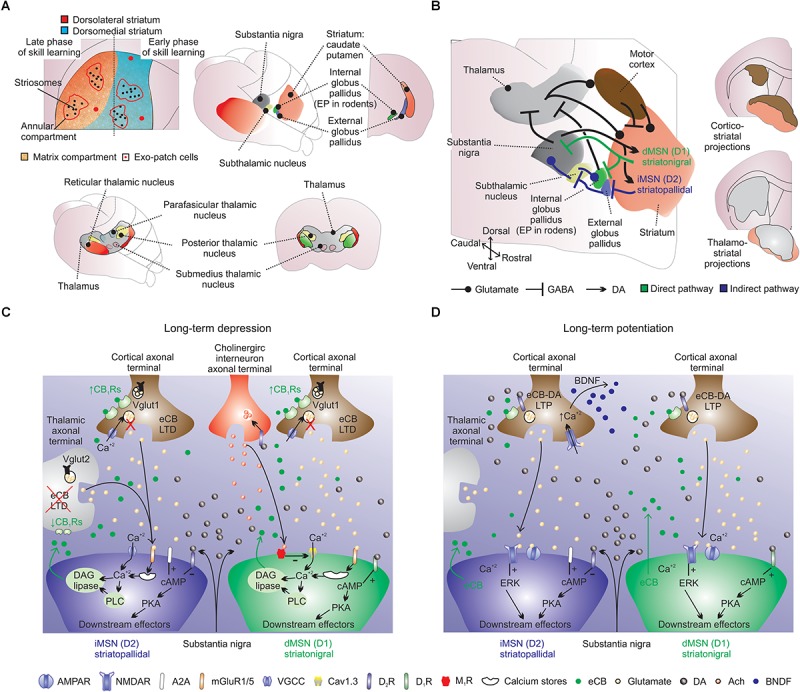
Striatal cellular and synaptic organization. **(A)** Striatum is a single structure in rodents: see striatal theoretical compartmentalization into dorsolateral (red; putamen) and medial (blue; caudate) striatum (Hjornevik, 2007; Heilbronner et al., 2016). Early and late skill learning are suggested to occur in a mediolateral fashion, respectively. (**A**, lower panels) In rodents the main thalamo-striatal afferents originate from the parafascicular nucleus. **(B)** The striatal circuit is composed of several intertwined structures involved in inhibition (indirect-pathway) or disinhibition (direct-pathway) of movement. The indirect-pathway (blue arrows) is formed by striatopallidal MSNs (iMSNs) that project to GABAergic pallidal neurons (external globus pallidus), which exert a powerful inhibitory control into proximal dendrites of glutamatergic neurons in the subthalamic nucleus. Subthalamic nucleus neurons send excitatory afferents to inhibitory output neurons of substantia nigra, and also to internal globus pallidus neurons. The net effect of indirect-pathway activation is inhibition of thalamo-cortical projection neurons, which can reduce cortical premotor drive and inhibit movement. The direct-pathway circuit (green arrows) is formed by striatonigral medium spiny neurons (dMSNs) that provides mainly inhibitory inputs to both GABAergic and dopaminergic cells in substantia nigra, which in turn sends axons to motor nuclei of the thalamus. Direct-pathway activation results in disinhibition of excitatory thalamo-cortical projections, resulting in activation of cortical premotor circuits and the selection/facilitation of movement. Glutamatergic striatal inputs from all cortical areas massively converge into the striatum. Also note the long-range GABAergic projections from the motor cortex to the dorsal striatum. Cortico-striatal and thalamo-striatal glutamatergic inputs target MSNs, large cholinergic interneurons and fast spiking interneurons. Cortico-striatal projections receive several inputs and integrate this information into striatal target neurons. Cholinergic interneurons receive scattered excitatory innervation mainly from thalamus and inhibitory synapses from MSNs. ‘Up-states’ are modulated by intrastriatal acetylcholine (ACh) and to strong intra-striatal DA release, D_1_Rs activation and striatal LTP. Midbrain dopaminergic terminals release dopamine (DA), which exerts a massive effect on all striatal cells. “Down-states” are associated to reduced intra-striatal DA, D_1_Rs/D_2_Rs activation and striatal LTD. Striatal LTP/LTD is also dependent on NMDARs activation and Ca^2+^ influx in MSNs. (**B**, right panels) Also note the distribution of cortico-striatal and thalamo-striatal afferents (Hunnicutt et al., 2016). **(C)** Long-term depression is dependent on mGLU_1/5_Rs, D_2_Rs, M_1_Rs, and C_1_BRs. For example, in iMSNs, prolonged stimulation of excitatory afferents paired with post-synaptic depolarization triggers the production and release of eCBs (e.g., 2AG) from the precursor diacylglycerol (DAG) through the activation of mGlu_1/5_Rs and phospholipase C (PLC; this process is dependent on Ca^2+^). **(D)** Long-term potentiation is dependent on NMDARs, D_1_Rs, A2ARs and CB_1_Rs. LTP is NMDARs dependent and is likely to involve the exocytosis of AMPA receptors. For example, LTP in the indirect pathway is negatively regulated by D_2_Rs (dependent on extracellular regulated kinase; ERK) and positively regulated by adenosine A2ARs.

The striatum is the main input nucleus of the basal ganglia and is a single structure in rodents. In rodents, striatal dorsolateral and medial portions are equivalent to putamen and caudate in primates, respectively (Heilbronner et al., 2016). The striatum can be divided in two compartments based on neurochemical characteristics and connections: patch (μ opiate receptor, substance P and enkephalin) and matrix (ACh esterase and calcium binding protein) (for references see Johnston et al., 1990; Gerfen, 1992; Lopez-Huerta et al., 2016). Anatomically, patch is a structure of interconnected tubes with finger-like branches and matrix is composed of well demarcated “dots” or “islands” of moderate to strong DA fluorescence (Olson et al., 1972; Johnston et al., 1990; Lopez-Huerta et al., 2016). Another recently added compartment is the annular compartment, which surrounds the striosome (or patch) (Brimblecombe and Cragg, 2015; Perrin and Venance, 2019). Matrix contains both direct and indirect striatal output pathways and does not exchange synaptic information with patch cells (Lopez-Huerta et al., 2016). Neurons of the matrix compartment make up about 85% of striatal volume (Johnston et al., 1990), contain the main outputs to globus pallidus and substantia nigra and are suggested to participate in behaviors associated with striatal and cortico-striatal function, such as skill learning (Lopez-Huerta et al., 2016). The patch (or striosome) compartment comprises a maximal striatal area of ≈15% of the rostral striatum in adult rodents (Lança et al., 1986). Patch and matrix compartments integrate limbic and sensorimotor information, through patch and exo-patch neurons (Smith et al., 2016) (Figure 3A). This compartmentalization affects DA release among striosomes (increase), annular compartment (decrease), and matrix (unmodified) (Brimblecombe and Cragg, 2015; Salinas et al., 2016; Perrin and Venance, 2019). In addition, cannabinoid receptor type 1 (CB_1_R) protein displays elevated expression in striosomes relative to the surrounding matrix (Davis et al., 2018). This complex striatal system is involved in motor skill learning in a medial (early skill learning) to lateral (late skill learning) gradient (Yin et al., 2009) (Figure 3A).

### Input Stage

The striatum is the input structure of the basal ganglia network. Cortical and thalamic glutamatergic signals converge into the striatum and are modulated by dopaminergic signals from mesolimbic nuclei (for references see Calabresi et al., 1996; Cerovic et al., 2013). In rodents, while cortico-striatal afferents are from several cortical regions, thalamo-striatal afferents are mainly from the parafascicular thalamic nucleus (Calabresi et al., 1996; Smith et al., 2014). Thalamo-striatal afferents have been implicated in controlling presynaptic suppression of cortico-striatal inputs through cholinergic interneurons, with implications on attentional shifts and cessation of ongoing motor programs (Ding et al., 2010). Recently, GABAergic inputs from the motor cortex to the dorsal striatum were described and implicated in motor control (Melzer et al., 2017). Optogenetic stimulation of these GABAergic long-range projections, such as M1 parvalbumin (PV)^+^ and M2 somatostatin (SOM)^+^, reduced locomotion (Melzer et al., 2017). These above-mentioned examples challenge the view of how thalamo- and cortico-striatal projections can modulate motor behavior and motor learning. Interestingly, post-stroke changes of cortical inhibitory markers, such as periinfarct PV^+^ and SOM^+^ have been reported (Zeiler et al., 2013; Alia et al., 2016; Spalletti et al., 2017), suggesting a role of this novel pathway for stroke recovery.

The majority of striatal cellular content is of GABAergic projection neurons, medium spiny neurons (MSNs), which are ≈95% of all neurons in the rat striatum (Kemp and Powell, 1971; Gerfen, 1992; Tepper et al., 2007; Kreitzer, 2009). The remaining glutamatergic afferents target intrastriatal interneurons: large cholinergic aspiny neurons (or cholinergic tonically active neurons), GABAergic PV^+^ or neuropeptide Y^+^/SOM^+^ interneurons (Tepper et al., 2004; for reviews and references see Kreitzer, 2009; Lovinger, 2010).

Cholinergic interneurons are only ≈1% of striatal cells, but their influence is significant due to large cell bodies and widespread axonal connections with MSNs. They receive scattered excitatory innervation mainly from thalamus and to a lesser extent from cortex, and inhibitory synapses from MSNs (Kreitzer, 2009 for references). Striatal cholinergic neurons may regulate functions of motor behavior and can release ACh, or can also co-release glutamate with ACh (Lim et al., 2014).

Motor cortex cortico-striatal afferents to patch and matrix compartments are mostly from layer V and to a lesser extent from layers II/III and VI (Smith et al., 2016). Differences in innervation of patch and matrix by cortical layers were reported, where patch and matrix compartments would receive cortico-striatal projections mostly from layers V/VI and superficial layer V and layer II/III, respectively (Gerfen, 1989). Later, the somatosensory cortex was considered to project exclusively to the matrix compartment, and layers Vb–VI preferentially to patches whereas layers III–Va to matrix axons (Kincaid and Wilson, 1996). But recent findings using genetic-based dissection suggest that cortico-striatal connections target patch and matrix compartments equally, regardless of region. Specifically, in M1, striatal patch/matrix inputs originate at layer V (≈75%), at layers II/III (≈10%) and at layer VI (≈15%). Either striosomes (or pathes) and matrix contain MSNs from direct and indirect pathways (Cerovic et al., 2013).

### Output Stage: Direct and Indirect Pathways

Activation of MSNs results in GABA release to the principal MSNs projections: external/internal globus pallidus and substantia nigra (Gerfen, 1992) (Figure 3B). The striatal cellular mechanisms provide a continuous balance between direct and indirect pathways. The direct pathway releases movements by disinhibiting thalamic activity and the indirect pathway restrains movements by inhibiting thalamic activity (Gerfen, 1992; Graybiel, 1995).

The direct-pathway circuit is formed by striatonigral MSNs (dMSNs) that provides mainly inhibitory inputs to both GABAergic and dopaminergic cells in substantia nigra, which in turn send axons to motor nuclei of the thalamus (Gerfen, 1992; Kreitzer and Malenka, 2008). Direct-pathway activation results in disinhibition of excitatory thalamo-cortical projections and activation of cortical premotor circuits to select/facilitate movement (Kreitzer and Malenka, 2008). The indirect-pathway is formed by striatopallidal MSNs (iMSNs) that projects to external globus pallidus GABAergic neurons (entopeduncular nucleus in rodents; EP); which exert a powerful inhibitory control into proximal dendrites of glutamatergic neurons in the subthalamic nucleus (Smith et al., 1990; Gerfen, 1992; Kreitzer and Malenka, 2008). Subthalamic nucleus neurons send excitatory afferents to inhibitory output neurons (i.e., substantia nigra) and the net effect of indirect-pathway activation is inhibition of thalamo-cortical projection neurons, which can reduce cortical premotor drive and inhibit movement (Kreitzer and Malenka, 2008) (Figure 3C). An intertwined function of both striatal pathways was recently proposed and postulated that the striatum would “filter” movement output integrating cortical glutamatergic and nigral dopaminergic inputs (Cui et al., 2013; Calabresi et al., 2014; Wang et al., 2015).

Cellular and synaptic organization is drastically changed in the striatum following stroke. It is likely that the striatal ‘search’ for different functional actions is accompanied by many structural changes. The bulk of the findings reviewed here supply abundant evidence of *where* these structural changes occur. For example, cortical lesions induce axonal sprouting in the denervated striatum (Uryu et al., 2001), dense network changes of crossed cortico-striatal projections (contralesional to ipsilesional; Napieralski et al., 1996), increased MSNs dendritic lengths (Gonzalez and Kolb, 2003), increased cortico-striatal projections and enkephalin mRNA levels (Napieralski et al., 1996; Uryu et al., 2001). Nevertheless, the post-stroke striatal ‘search’ task for functional actions is still poorly understood, such as *how* the striatal network would “filter” movement output integrating these novel and compensatory striatal plasticity, e.g., crossed cortico-striatal projections. Crossed cortico-striatal projections are only ≈20% of the total afferents to the contralateral striatum (Smith et al., 2016), but massive collateral sprouting from this minor projection would still be representative (Uryu et al., 2001). Interestingly, crossed cortico-striatal neurons preferentially make synapses with dMSNs, while cortico-striatal neurons with iMSNs (Cowan and Wilson, 1994; Florio et al., 2018). This suggests that the increased post-stroke crossed cortico-striatal inputs would favor movement disinhibition in the ipsilesional motor system. This can modulate excitatory outputs to thalamic neurons in the ipsilesional hemisphere that are the final pathway from the striatal system at the output level.

### Endogenous Plasticity Mechanisms: “Up-” and “Down-States”

The striatum exhibits anatomo-functional complexity and intrinsic diversity, such as synaptic transmission may depend on bidirectional plasticity and spike-timing dependent plasticity (STDP). This implies that the precise relative timing and interval between presynaptic and postsynaptic action potentials determine the strength of striatal synaptic potentiation or depression (Cerovic et al., 2013; Perrin and Venance, 2019). Albeit, the role of such type of plasticity for LTP/LTD is still under debate (Lisman and Spruston, 2010). This is a very complex subject, plasticity mechanisms are variable and depend on many factors, such as: stimulation protocol, slice preparation, interplay between receptors, among others. Here, we show a simplified view of striatal plasticity mechanisms, focused on the most recent findings that relate to motor skill learning. The striatum acts as a relay nucleus, which integrates strong cortical and thalamic inputs and retransmit information via indirect thalamic projections. As aforementioned, motor skill learning modifies striatal responses during this relay process, and these changes are suggested to occur in a medial to lateral fashion (Tepper et al., 2007; Kreitzer, 2009; Yin et al., 2009). Hence, these changes in transmission affect the activity of thalamo-cortical projections and, as a consequence, motor behavior (Fisone et al., 2007). Long-lasting changes of cortico-striatal and thalamo-striatal synapses are considered to be a cellular basis of motor learning.

Striatal and thalamic reorganization following a cortical lesion are well documented. For example, Gonzalez and Kolb (2003) showed that small middle cerebral artery occlusion results in increased MSNs dendritic lengths in both hemispheres. Additionally, larger occlusions increased MSNs dendritic length in the contralesional hemisphere, but dendritic branching in the ipsilesional hemisphere (dorsolateral striatum; Gonzalez and Kolb, 2003). Interestingly, in the striatum a single EPSP from cortico-striatal glutamatergic fibers is not sufficient to depolarize MSNs to overcome the voltage-dependent Mg^2+^ blockade of NMDARs. But, in pathological conditions, such as stroke, a single excitatory input can lead to the activation of striatal NMDARs due to the “pathological” removal of the magnesium block (Calabresi et al., 2000 for references). Cortical ischemia also results in reduction of neurons, GABA_A_ receptors and increase in NMDARs in the ipsilesional thalamus projecting to cortical damaged areas (Qu et al., 1998).

Thus, stroke disrupts cortico-striatal glutamatergic inputs in the damaged site, but also induces MSNs plasticity in both hemispheres. Glutamate has an important role on regulating striatal excitability, such as in response to glutamatergic synaptic input MSNs can transition to a depolarized “up state” near spike threshold. Short-term changes on “up-state” potentials involve KCNQ channels modulated by intrastriatal cholinergic interneurons. ACh binds to m_1_Rs in MSNs and activate KCNQ channels through PLCβ/PKC pathway resulting in increased MSNs excitability (Shen et al., 2005). “Up-states” are also suggested to be linked to strong intra-striatal DA release, D_1_Rs activation and striatal LTP.

Conversely, “down-states” are associated to low intra-striatal DA, D_1_Rs/D_2_Rs activation and striatal LTD (Calabresi et al., 1997, 2007; Shen et al., 2008). Striatal LTD reduces the activity of projecting GABAergic MSNs, and influences the output signals from the striatum to other structures that control motor activity (Calabresi et al., 1992). Skill learning and cortico-striatal LTD in the dorsolateral striatum are dependent on AC5 and cAMP signaling (Kheirbek et al., 2009). In addition, the generation of striatal LTD requires Ca^2+^ influx through voltage dependent Ca^2+^ channels, Ca^2+^-dependent protein kinases (Calabresi et al., 1994) and synthesis of endocannabinoids (eCBs) (Cerovic et al., 2013). eCB signaling integrates signals from different neurotransmitters, such as glutamate and dopamine, with voltage gated Ca^2+^ signals (Cerovic et al., 2013). eCB-LTD is modulated by D_2_Rs and dependent on postsynaptic mGluRs activation and L-type calcium channels (Kreitzer and Malenka, 2005). Similarly glutamate-LTD is also modulated by D_2_Rs. For example, after high-frequency stimulation of cortico-striatal fibers, mice lacking D_2_Rs shift from the expected LTD to a NMDARs-mediated LTP (Calabresi et al., 1997). Another interesting feature of striatal eCB modulation is the differential role on cortico-striatal (high [CB_1_Rs]) and thalamo-striatal (low [CB_1_Rs]) afferents (Wu et al., 2015). The existence of two forms of striatal LTD induced at up- and down-states (Mathur et al., 2013) and bidirectional DA modulation of eCB-LTD expression (Cui et al., 2015, 2016; Wu et al., 2015; Xu et al., 2018), reflects the complex interactions involved in striatal action control.

Skill learning activates the cortico-striatal pathway, the glutamatergic system and complex cellular mechanisms related to NMDARs activation. In rotarod trained animals, the striatal NMDARs subunit NR1 is up-regulated (D’Amours et al., 2011) and NMDARs or NR2A blockade impairs motor learning in this task in a dose-dependent manner (Lemay-Clermont et al., 2011). Additionally, Yin et al. (2009) demonstrated the medial to lateral gradient of early and late skill learning, respectively, where LTP is observed in iMSNs at the dorsolateral striatum (late skill learning), but not at dMSNs; also that early skill learning plasticity is likely non-NMDARs dependent in the dorsomedial striatum (Yin et al., 2009). Recently, this view has been challenged by a parallel (associative: medial pre-frontal cortex and dorsomedial striatum; sensorimotor: M1 and dorsolateral striatum), but dissociable, processing in cortico-striatal inputs during skill learning (Kupferschmidt et al., 2017). Kupferschmidt et al. (2017) show parallel activity in these associative and sensorimotor circuits while mice refined rotarod performance. Additionally, thalamo-striatal NMDARs-LTD plasticity is also observed in iMSNs and dMSNs (Ding et al., 2008; Ellender et al., 2013; Wu et al., 2015), further that blocking serotonergic signaling favor spike-timing-dependent LTD in dMSNs (Cavaccini et al., 2018). Finally, that presynaptic NMDARs are also involved in cortico-striatal LTP plasticity through BDNF release (Park et al., 2014), but the role of this presynaptic plasticity to stroke relearning is still underexplored. Briefly, abundant findings support the idea that skill learning experience produces changes in cortico-striatal transmission efficacy and induce the formation of sensorimotor links (see suggested reviews at the end of this section). For example, specific context-dependent patterns of cortical activity can engage selected motor programs (Mahon et al., 2004), and such changes partially depend on striatal LTP/LTD mechanisms.

State changes are also involved in striatal plasticity, such as repetitive cortico-striatal transmission during the “up-state” can overcome the threshold for NMDARs activation and, if associated with a strong release of DA, lead to LTP induction. Conversely, repetitive transmission during the “down-state,” in association with low DA levels should lead to LTD (Calabresi et al., 1997, 2007; Charpier and Deniau, 1997). The circuitry of relevant motor programs would undergo plastic changes through induction of striatal LTD and LTP (Reynolds et al., 2001). Excitation of dMSNs results in the disinhibition of premotor networks, thus LTP at excitatory striatal inputs would be favorable to the initiation of movements and critical for motor learning (Charpier and Deniau, 1997; Yin et al., 2009; O’Hare et al., 2016).

Striatal cellular mechanisms involved in synaptic modulation are mainly related to presynaptic inhibition of neurotransmitter release through GPCRs (via G_i/o_) and eCBs (Lovinger, 2010; Cerovic et al., 2013). Cortico-striatal terminals are controlled by presynaptic receptors, when activated can result in negative feedback on the striatal release of glutamate (Calabresi et al., 1996). As aforementioned, activation of presynaptic CB_1_Rs may have distinct effects on cortico-striatal and thalamo-striatal axonal terminals, due to differential presynaptic CB_1_R expression of these inputs (Wu et al., 2015) (Figure 3C,D). Finally, it is important to mention the role of striatal fast-spiking interneurons for experience-dependent behavior (O’Hare et al., 2018) and striatum-dependent sequence learning (via feedforward inhibition that restricts MSN bursting and calcium-dependent synaptic plasticity) (Owen et al., 2018).

Here, we highlight the main and most up-to date striatal plasticity mechanisms. This is currently a hot topic in neuroscience and several recent reviews explore this subject in depth. For example, on how (1) dopamine neurotransmission acts in concert with several neurotransmitters to regulate cortical, thalamic and limbic excitatory inputs (Surmeier et al., 2009; Bamford et al., 2018); (2) are the complex interactions between striatal plasticity and learning (Perrin and Venance, 2019); (3) are the complex computations performed by the basal ganglia circuits (Yin, 2017); (4) to clarify the relationship between neuronal plasticity in the basal ganglia and habitual behavior (with focus in kinematics of movement; O’Hare et al., 2018); (5) eCB-DA interaction affects striatal synaptic plasticity (Mathur and Lovinger, 2012); (6) genetic tools enabled new experimental protocols to reveal striatal cell types and connectivity (Silberberg and Bolam, 2015); (7) the thalamo-striatal system changes in diseased states (Smith et al., 2014). Perrin and Venance (2019) suggest that a new period of abundant and constructive debates is opened in the field of striatal plasticity. We suggest that the post-stroke recovery field should take advantage of this fruitful period to establish new mechanisms and therapies for stroke recovery.

## Two Phases of Stroke Recovery – ‘Fast’ and ‘Slow’ Motor Relearning

Cortical and striatal circuits work together during the development of motor skills characterized in two phases. A ‘fast’ improvement of motor performance with rapid behavioral outcomes, which can be observed both within a single training session and across the first few sessions. And a ‘slow’ improvement that develops across sessions, with more moderate gains in performance that progress across multiple training sessions (Karni et al., 1998; Kleim et al., 2004). During ‘fast’ motor learning cortical and striatal circuits undergo rapid and extensive recruitment with parallel activity; conversely, during ‘slow’ learning the activity patterns differs between structures (Costa et al., 2004). This suggests that both structures work to rapidly adapt the motor system to the new task. During ‘slow’ learning parallel recruitment is less often and cortical or striatal recruitment are likely associated to distinct movement features (Costa et al., 2004).

In the same line of thoughts, cortical motor representation changes do not contribute to the initial acquisition of motor skills – but represent the consolidation of motor skills that occur during the later ‘slow’ phase of learning (Kleim et al., 1998; Kleim et al., 2004; Krakauer and Shadmehr, 2006). This suggests that ‘fast’ skill improvement is related to an ongoing process, not yet consolidated intra-cortically, thus dependent on parallel striatal activity. For example, post-stroke relearning could activate a large number of cortical cells during initial stages, but with practice increase the number of cells active that correlate with the motor task (as for motor learning; Peters et al., 2014; Makino et al., 2016). This would translate in a functional motor map/engram if sufficient reinforcement of motor action render behavior habitual (Hosp and Luft, 2011).

In other words, if during this striatal ‘search’ task the rewiring does not elicit functional movements, the reward-related reinforcement is poor. Hence, this dysfunctional motor map should not consolidate and the striatal ‘search’ should continue. Recovery would rely on this parallel cortico-striatal processing until permanent and relevant changes are reinforced and stored intra-cortically. In search for spared/functional movements the striatal network attempts to integrate the remaining pieces of cortico-thalamic network. Callosal cortico-cortical connections from the contralesional homotopic motor cortex and crossed cortico-striatal connections help to direct and guide this changing network - in search for functional movements. In addition, thalamo-cortical loops relayed by the striatum have the flexibility needed for adaptation to imposed behavioral demands following motor skill training (Biane et al., 2016). Striatal ‘up’ and ‘down’ states modulated by changed glutamatergic inputs – in some regions reduced (lesion) and in others increased (compensatory connections), redirects information flow throughout the circuits. Hebbian plasticity in the associated population of cells may change synaptic strengths to favor plasticity of pathways coincidently active and eventually results in a refined ensemble and stereotyped functional behavior (Makino et al., 2016). Thus, remainings of previous loops that are disrupted by the lesion can join this novel network assisted by intense cortico-striatal parallel processing – a novel cortical representation is later formed. This idea is supported by recent findings on how optogenetic rewiring of thalamo-cortical circuits can restore function in the stroke injured brain (Tennant et al., 2017). Rehabilitation would offer the repeated opportunity to explore, select and refine impaired movements (Makino et al., 2016).

Interestingly, post-stroke, a relatively ‘fast’ behavioral motor recovery is followed by a plateau of ‘slow’ or absent recovery (62–70% proportional to the initial impairment; Jeffers et al., 2018). Rehabilitation is thought to help guidance and pruning of new connections, which due widespread activation of growth and plasticity mechanisms following the lesion may form unbalanced, non-functional connections (Murphy and Corbett, 2009). This would fit perfectly with the striatal ‘search task’ during relearning, adding specificity and homeostatic balance to novel functional connections. Indeed, early onset of rehabilitation during this ‘fast’ period consistently results in better functional outcomes (Biernaskie et al., 2004; Murphy and Corbett, 2009).

At chronic phases of ‘slow’ motor recovery the motor circuits would constantly modify to select and refine novel skills. How this process occurs is still unclear, it is likely that the ‘fast’ attempt to recover function involves the old cortical/striatal circuits, or what is left of it. It is reasonable to think that this is a less demanding homeostatic adaptation, compared to novel long-distance connections and changes of regions remote from the lesion – previously minimally involved in the motor skill.

**FIGURE 4:**
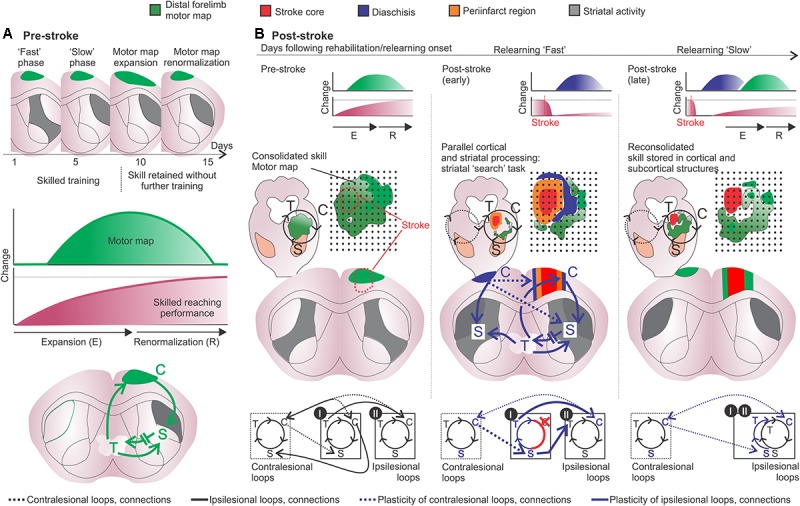
Motor learning and post-stroke relearning. (**A**, upper and middle panels) Motor learning is divided into a fast phase, with rapid improvement in performance, and a slow phase related to a “cortical learning mode” likely involved in the consolidation of motor skills. After 8–10 days of training the motor skill is acquired and transient changes imply in motor map expansion-renormalization according to the trained skill (Kleim et al., 2003; Molina-Luna et al., 2008; Wenger et al., 2017). (**A**, lower panel) The motor map/engram is composed by the motor map of the acquired skill and by specific cortico-striatal-thalamo-cortical loops (green). (**B**, left panels) From top to bottom, the respective motor map (green) is sustained by specific cortico-striatal-thalamo-cortical loops following motor map expansion-renormalization related to the acquired skill. The loop diagram describes the ipsilesional (black lines) and crossed (black hashed lines) connections from specific cortical, striatal and thalamic regions I and II (bottom). (**B**, middle and right panels) From top to bottom, post-stroke, diaschisis may affect motor representations with, respectively, impaired skilled function. The lesion core is surrounded by dysfunctional tissue, such as periinfarct tissue (orange) and regions of focal and non-focal diaschisis (blue). Connectivity is changed and the putative participation of adjacent cortico-striatal-thalamo-cortical loops in the ipsi- and contralesional hemispheres is shown. Cortical stroke (red cross and connections) induces cellular and plastic changes (blue arrows and letters). Post-stroke cellular and plastic changes during relearning of the skilled movements may also occur in a medial to lateral fashion, similarly to motor learning and may include: increased activity in the medial portion of the ipsilesional striatum (gray); increased activity of uncrossed corticospinal fibers and of mirror-image neurons in the contralesional hemisphere (blue lines); increased activation of crossed corticostriatal fibers (blue hashed lines); short- and long-term striatal plasticity that results in increased dendritic branching and spines in the ipsi and contralesional hemispheres; the putative participation of the contralesional medial striatum during later phases of slow relearning; motor maps are reorganized and potentiated corticospinal projections to the affected muscles are available, both in the ipsi and contralesional hemispheres (i.e., crossed and uncrossed corticospinal fibers) (Pruitt et al., 2016). Connections and loops are rearranged and the newly formed motor configurations are encoded into the lateral portions of bilateral striatum. C, cortex; S, striatum; T, thalamus.

Indeed, facilitation of LTP (1 week; Hagemann et al., 1998) and use-dependent neuronal activation (10 days; Clarke et al., 2014) in the dysfunctional perilesional cortex occur early after stroke and may indicate ‘fast’ changes of function in this region. Such changes are absent in homotopic contralateral areas at this early time point (Hagemann et al., 1998; Clarke et al., 2014). In chronic post-stroke, reorganization of functional circuits of parallel projecting cortical areas in the ipsilesional and contralesional hemispheres suggests that long-term or ‘slow’ reorganizational changes involve undamaged regions adjacent and distant to the lesion core (3–4 weeks; squirrel monkeys; Nudo et al., 1996b, 2001). Examples of such ‘slow’ changes include: damaged ipsilesional cortico-striatal connections that are functionally ‘replaced’ by increased crossed cortico-striatal projections to the denervated striatum (Cheng et al., 1998); axonal sprouting in the contralesional striatum (Uryu et al., 2001) that may be linked to increased crossed cortico-striatal projections (ipsilateral to contralateral; Napieralski et al., 1996; Uryu et al., 2001). These processes would slowly evolve during relearning of movements through striatal exploration, selection and refinement of functional movements. We suggest that these features would be part of a ‘slow’ compensatory relearning mechanism by which the motor function would be restored (Figure 4).

### Are Cortical Motor Maps a Reflection of Consolidation?

Here we discuss how the motor map may reflect consolidation–reconsolidation processes. If the motor map is a reflection of consolidation of motor memories it should stabilize and persist over time. Evidence, both in humans (Ward et al., 2003; Tombari et al., 2004) and rodents (Molina-Luna et al., 2008; Reed et al., 2011), indicates that this is indeed not the case. According to the expansion-renormalization theory, learning-related neural processes often follow the sequence of expansion, selection and renormalization (reviewed in Wenger et al., 2017). Motor map expansion may be thought as a transitory “cortical learning mode”, which according to the expansion-renormalization model should subsequently refine and compact, maintaining the readiness of the learned skill (Molina-Luna et al., 2008; Wenger et al., 2017). It is reasonable to think that following motor skill learning prolonged map expansion may reduce the flexibility needed to acquire subsequent novel motor skills.

Nevertheless, several studies indicate that the motor map may encompass some features of consolidated motor memories, such as synaptogenesis (Kleim et al., 2004), susceptibility to protein synthesis inhibition (anisomycin, ANI; Kleim et al., 2003), dependence of DA (following intrastriatal 6-hydroxydopamine injections; Brown et al., 2009 – also see Hosp and Luft, 2013) and influence of cholinergic (Conner et al., 2005; Ramanathan et al., 2009) and serotonergic (Scullion et al., 2013) mechanisms. It is also the case of post-stroke motor maps, rehabilitative training may drive the reemergence and reorganization of motor maps (Nudo et al., 1996b; Conner et al., 2005; Ramanathan et al., 2006) and infusion of ANI can decrease the reorganized motor map, synaptic density and post-stroke performance improvement (Kim et al., 2018).

Similarly, studies support the role of the motor cortex for skilled behavior (Guo et al., 2015; Miri et al., 2017; Galiñanes et al., 2018), for motor learning (Peters et al., 2014) and of the somatosensory cortex for motor memory update and motor adaptation (Mathis et al., 2017). Conversely, evidence also indicates that non-dexterous motor performance is not dependent on the motor cortex (Kawai et al., 2015; Miri et al., 2017; also see Papale and Hooks, 2018). According to systems consolidation theory, at remote points the memory trace should depend more on cortical areas rather than subcortical regions (Hardt and Nadel, 2018). It is plausible to think the motor map expansion as the learning mode that further consolidate the motor memory in the complex cortico-striatal-thalamo-cortical loops. Further studies should demonstrate the role of striatal and thalamic lesions on motor maps following learned skilled behavior and in non-dexterous motor controls. In non-trained animals, motor maps are not affected by either medial or lateral striatal lesions, suggesting that motor impairments after such lesions are not simply related to motor map alterations (Karl et al., 2008). It is still to be answered if this is the case for skilled trained animals, and also for post-stroke reemergence and reorganization of motor maps. In a recent study, it was shown that striatal lesions are important for spontaneous recovery of non-skilled tasks (i.e., cylinder task) but not for dexterous reaching behavior (i.e., staircase task) (Karthikeyan et al., 2018). This is in accordance with the emerging consensus on the concept of cortical control over skilled motor behavior (Papale and Hooks, 2018).

Does motor map size really matter for consolidated motor skills? We suggest that future studies should focus more on the complexity and quality of the motor output, not strictly the size, given the above-mentioned dynamic nature of motor map size on short time scales (Molina-Luna et al., 2008; Reed et al., 2011). One suggested possibility is that M1 outputs captured by motor mapping may be necessary for driving plasticity in downstream structures or for initiating consolidation processes (Papale and Hooks, 2018). This consolidation would take place somewhere along the cortico-striatal-thalamo-cortical loops. The reflection of such consolidation should not always relate to increased size of motor representations, but to its efficiency in driving functional combinations of movements. For example, motor maps can be categorized in complex multiplanar movements such as abduction and adduction (Harrison et al., 2012; Bonazzi et al., 2013). Additionally, post-stroke emergence of abnormal movements or synergies in rats suggests that relearning may involve motor map reorganization to generate functional control of such complex movements (Balbinot et al., 2018). In our opinion this may not always be reflected by a greater size of motor representation, but to its content, such as the combination of different cortical modules for efficient post-stroke compensation.

## Diaschisis as a Consolidation-Reconsolidation Process

von Monakow’s theory of diaschisis describes neurophysiological changes distant to a focal brain lesion (von Monakow, 1914; Carrera and Tononi, 2014). Accordingly, this concept posits that functional changes in brain structures remote from the site of a focal brain damage can underlie functional recovery following stroke (Witte, 1998; Seitz et al., 1999). Diaschisis can be subdivided into two types: (1 – focal diaschisis) reduction of energy metabolism at rest or during activation in anatomically intact brain regions distant from the lesion; (2 – non-focal diaschisis) change in coupling between two regions of a defined network or connectome involving areas distant from the lesion (Carrera and Tononi, 2014). For example, ipsilesional thalamic diaschisis is characterized by thalamic hypoperfusion in the acute phase of stroke patients (Reidler et al., 2018). Ginsberg et al. (1989) showed evidence of functional thalamic diaschisis following small thrombotic infarct in rats-in the form of impaired thalamo-cortical activation, in other words, thalamic activation was normal at rest but failed to exhibit the expected increment in response when stimulated. Similarly, Carmichael et al. (2004) described acute hypometabolism in a broad region of cortex adjacent to the stroke, striatum and thalamus – not related to cerebral blood flow reduction or reperfusion injury. At chronic stages, the ipsilesional cortical diaschisis still encompassed an area ≈13 times larger than the infarct (Carmichael et al., 2004). The authors suggest that the overlap of axonal sprouting and cortical hypometabolism are likely related to a process of neuronal reorganization and reconnection following stroke (Carmichael et al., 2004). Interestingly, functional diaschisis may involve reduced activation of some areas, but increased responsiveness of others (Carrera and Tononi, 2014). Suggesting that diaschisis is more than simple loss of function, but changed function of areas distant to the lesion.

Is diaschisis a cause or consequence of this process of neuronal reorganization and reconnection? It is interestingly how diaschisis occurs early following stroke and that rehabilitation optimally affect behavioral outcome also at this early time point. It is likely that diaschisis is a gross, brain wide process of reorganization, much less subtle than a reconsolidation process, for example. Albeit different, this neuronal reorganization and reconnection must involve the classical and well described mechanisms of consolidation and reconsolidation of motor memories. Motor map/engram integrity requires continuous expression of specific proteins, such as local injections of protein synthesis inhibitors (e.g., ANI, cycloheximide) results in loss of movement representations (Kleim et al., 2003). A possibility is that motor maps are constitutively plastic, its existence relies upon constant presence of specific neural signals (Monfils et al., 2005). Once stroke disrupts these specific neural signals to distant regions, the related regions change functionality. Thus, it is reasonable to think that diaschisis would not be a cause, but a consequence of neuronal reorganization and reconnection processes.

Given the aforementioned positive role of rehabilitation during this early period of changed function, it is possible that learning mechanisms can tailor this neuronal reorganization and reconnection process. In the light of the motor learning school, this process of neuronal reorganization and reconnection could be described as a process of ‘rebuilding’ the motor engram/map to regain function - such as during consolidation/reconsolidation of motor memories.

**FIGURE 5:**
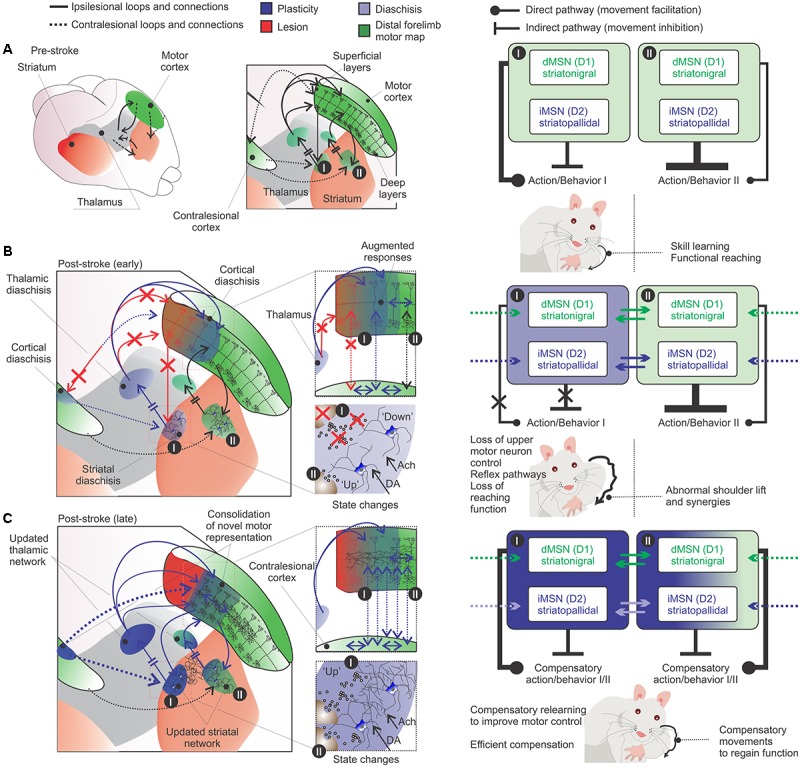
Diaschisis as a consolidation-reconsolidation process. (**A**, left) Movement before stroke involves the selection of the appropriate motor program for the specific action (cortico-striatal-thalamo-cortical loops). (**A**, right) Specific actions are linked to specific cortico-striatal-thalamo-cortical loops. This specific functional region may control a voluntary movement, using the appropriate motor sequence for a coordinated muscular action. (**B**, left panels) Following stroke, diaschisis of regions close or distant to the infarct core (light blue) affects the functionality of the motor network and disrupts or change the specific action (red arrows: lost connections; blue and black arrows: remaining connections). (**B**, right panels) This results in loss of upper motor neuron control over voluntary movements and the emergence of abnormal movements (Balbinot et al., 2018). Compensatory relearning is unlikely to fully restitute movements of the paretic limb, which should retain some of the abnormalities and deficits in the specific action. Functional compensatory movements may be reinforced by lateral inhibition between ipsilesional MSNs (solid lines; the striatal region I influence striatal region II) and/or contralesional cortico-striatal connections (hashed lines). This reinforcement is shaped by striatal state changes and cortical plasticity. **(C)** Rehabilitation provides the recollection of visual, tactile and motor cues: the motor output is a changed action tailored over many rehabilitation sections during the “striatal search task” and the “cortical learning mode”. It is likely that consolidation–reconsolidation mechanisms are slowly acting to shape these circuits during rehabilitation. Compensatory brain regions may supplement function of damaged areas and a novel motor engram is formed (dark blue) (e.g., Kim et al., 2018). The system is shaped toward the specific actions used over rehabilitation sections. Hence, the generation of a novel motor engram is supported by a series of adjustments in connections of the redundant motor system network. Right panels in **(A–C)** are inspired by a new perspective for striatal local circuitry plasticity (Burke et al., 2017). The authors explore how lateral inhibition (between MSNs) can contribute to the formation of functional units to process, integrate and filter inputs to generate motor patterns and learned behaviors (Burke et al., 2017).

Indeed, previously consolidated motor memories can undergo a labile state upon reactivation. The reactivated motor memory can be modified through reconsolidation (Walker et al., 2003; Censor et al., 2010; de Beukelaar et al., 2014, 2016). If this is the case, rehabilitation would promote such recollection of visual, tactile and spatial cues to provide cortical and striatal systems with relevant information to rebuild the motor engram. Relearning should involve the reconsolidation of previous motor loops that were not completely destroyed by the lesion, they are updated. For example, in remote regions that undewent diaschisis following stroke. Additionally, consolidation of novel compensatory motor engrams – novel connections to compensate for the loss of tissue and motor network function (Figure 5).

Abundant evidence of learning-like plasticity at both, close and remote regions, have been reported following stroke. For example, axonal sprouting within local projections, intra-cortical projections, long distance interhemispheric projections and cortico-striatal projections (Wilson, 1987; Lévesque et al., 1996; Carmichael and Chesselet, 2002; Carmichael, 2003). These cortical plastic changes are supported by the induction of growth-promoting genes (Brown and Murphy, 2007), also induced by motor learning (Cheung et al., 2013; Hertler et al., 2016). Striatal gene expression also occurs after a new complex motor task is memorized and most of the upregulated genes are associated with synaptic plasticity (D’Amours et al., 2011). Overall, the permissive environment following stroke leads to cortical rewiring (Winship and Murphy, 2008) and this phenomenon may be experience- and time-dependent. This suggests that plasticity, experience- and time-dependence are common attributes of both stroke recovery and consolidation-reconsolidation processes. In addition, recent evidence shows that the classic molecule CREB, involved in many learning processes, controls cortical circuit plasticity and functional recovery following stroke (Caracciolo et al., 2018). These learning-like plastic changes may relate to consolidation-reconsolidation of ‘novel’ or ‘updated’ motor engrams/maps.

Rehabilitative experience may slowly shape these new connections in a constant process of consolidation-reconsolidation of motor memories. Given the complex and continuous nature of the rehabilitation stimulus, these processes would evolve and change continuously. In other words, the consolidation-reconsolidation process of motor memories would change throughout multiple trials. This is more challenging to understand compared to declarative or fear memories that often are treated as a single trial event. Despite these differences, the understanding that recovery/relearning of movements may share the same classic mechanisms involved in learning and memory opens a new venue of timed and focused interventions during rehabilitation. For example, the timed use of learning enhancing drugs before, during or after rehabilitation sections. Or, use of protein synthesis inhibitors upon reactivation of maladaptive motor programs, such as dysfunctional compensation or learned bad-use.

## Conclusion

Optimization of stroke recovery focused on learning mechanisms should follow the same logic of previous learning and memory studies. The fact that the motor skill redevelops slower, across multiple trials, presents a challenge for preclinical studies on the mechanisms of post-stroke compensatory relearning (Schubring-Giese et al., 2007). Recovery following stroke is related to rewiring at many corticospinal tract regions and requires *upstream* cortical commands (Lindau et al., 2014; Wahl and Schwab, 2014; Wahl et al., 2014). The deep cortico-striatal network plays a pivotal role on the selection of actions (Arber and Costa, 2018) and a putative role on selection of compensatory actions following stroke. Cortico-thalamo-cortical loops are relayed by the striatal network that is drastically changed following stroke. The denervated striatum receives increased crossed cortico-striatal connections and undergo plastic changes. In search for functional action, the striatal network participates on the reorganization of the motor system and uses spared and compensatory motor networks. As the system reorganizes, rehabilitation should induce functional compensatory movements (large lesions) or full restitution of functional movements (smaller lesions) (Murphy and Corbett, 2009; Jones, 2017). Rehabilitation therapies should focus on *how* to improve relearning.

Several cortical and striatal cellular mechanisms that influence motor learning may also influence post-stroke compensatory relearning, such as: mGlu_5_Rs agonists (Homayoun et al., 2004), 5-HT reuptake inhibition (McCann et al., 2014), cholinergic system manipulation to induce plasticity (Ramanathan et al., 2009; Conner et al., 2010) and improve post-stroke rehabilitation/relearning (Wang et al., 2016), dopaminergic manipulation (Ogura et al., 2005; Akita et al., 2006; Brown et al., 2009, 2011; Hosp et al., 2011; Kawashima et al., 2012; Hosp and Luft, 2013), selective manipulation of dorsolateral striatum matrix compartment (Lopez-Huerta et al., 2016), NMDARs manipulation in the dorsolateral striatum (Dang et al., 2006; Beutler et al., 2011; Lemay-Clermont et al., 2011) and modulation of FS interneurons in the dorsolateral striatum (Kao et al., 2015; Xu et al., 2016).

If the motor recovery process is directly shaped by cortical-striatal-thalamic interactions, whatever changes *downstream* must be secondary to this relearning process. For example, the importance of spinal cord (Lindau et al., 2014; Wahl et al., 2014) and red nucleus (Mosberger et al., 2018) plasticity for stroke recovery. Thus, optimizing relearning focusing on cortical-striatal-thalamic interactions is likely acting directly on the mechanism that induce learning-related plasticity both, *in site* and *downstream* to the lesion. Despite the importance of the striatum to learning, evidence of striatal participation on stroke recovery is lacking. In addition, the majority of basic stroke research studies does not fully address the role of striatal lesions and mechanisms (Edwardson et al., 2017).

*In vivo* recordings have changed the way we think about motor learning (Costa et al., 2004), motor recovery (Ramanathan et al., 2018) and sensorimotor representation plasticity following stroke (Harrison et al., 2013). The most pressing question facing researchers have evolved from *where* recovery occurs, to *how* it occurs. We suggest that unveiling *how* the recovery/relearning process occurs is like to take a walk on a well know path of motor learning. This path should remind us that neural activity and synaptic plasticity are constantly interacting, both at individual synapses and within neural circuits. Our challenge is to understand *how* they operate in a behaving brain to support post-stroke compensatory relearning.

## Author Contributions

GB wrote the manuscript and drawn the figures. CS reviewed the manuscript.

## Conflict of Interest Statement

The authors declare that the research was conducted in the absence of any commercial or financial relationships that could be construed as a potential conflict of interest.
